# Programmable design of isothermal nucleic acid diagnostic assays through abstraction-based models

**DOI:** 10.1038/s41467-022-29101-1

**Published:** 2022-03-28

**Authors:** Gaolian Xu, Julien Reboud, Yunfei Guo, Hao Yang, Hongchen Gu, Chunhai Fan, Xiaohua Qian, Jonathan M. Cooper

**Affiliations:** 1 Nano Biomedical Research Centre, Nano Biomedical Research Centre, Shanghai Jiao Tong University, Shanghai, 200030 China; 2 Division of Biomedical Engineering, James Watt School of Engineering, University of Glasgow, Oakfield Avenue, Glasgow, G12 8LT UK; 3 School of Chemistry and Chemical Engineering, Frontiers Science Center for Transformative Molecules, Shanghai Jiao Tong University, Shanghai, 200240 China; 4 School of Biomedical Engineering, Shanghai Jiao Tong University, Shanghai, 200240 China

**Keywords:** DNA, Assay systems, Medical and clinical diagnostics, Biomedical engineering

## Abstract

Accelerating the design of nucleic acid amplification methods remains a critical challenge in the development of molecular tools to identify biomarkers to diagnose both infectious and non-communicable diseases. Many of the principles that underpin these mechanisms are often complex and can require iterative optimisation. Here we focus on creating a generalisable isothermal nucleic acid amplification methodology, describing the systematic implementation of abstraction-based models for the algorithmic design and application of assays. We demonstrate the simplicity, ease and flexibility of our approach using a software tool that provides amplification schemes de novo, based upon a user-input target sequence. The abstraction of reaction network predicts multiple reaction pathways across different strategies, facilitating assay optimisation for specific applications, including the ready design of multiplexed tests for short nucleic acid sequence miRNAs or for difficult pathogenic targets, such as highly mutating viruses.

## Abstract (web summary)

Detecting nucleic acids often requires choosing between different amplification mechanisms. Here the authors present a generalisable and programmable isothermal methodology, demonstrated in clinical applications, including for multiplexed detection of short miRNAs.

## Introduction

Nucleic acid amplification has become the cornerstone in the design of novel, functional molecular assays, driven by the increasingly demanding analytical challenges associated with detecting complex biomarker targets for non-communicable or infectious diseases. Isothermal nucleic acid-based amplification assays remain critical in such diagnostics, allowing assays to be performed at the point-of-care, without the complex instrumentational demands of current gold-standard polymerase chain reaction (PCR) based assays.

Currently, the design and implementation of many new isothermal assays is based upon mechanisms involving steric and/or functional interactions between secondary structures of DNA strands^1,2^, which can be optimised iteratively to enable rapid nucleic acid analysis^3^. Mechanistically, such secondary structures lead to specific designated products, which are formed in the initial steps and are used as mediators in the subsequent amplification cycling steps.

Common examples, where the manipulation of such specific sequences forms secondary structures, include amplification systems based on hairpin constructs, used for example in cross priming amplification (CPA)^4^ and loop-mediated DNA amplification (LAMP)^5^. In such mechanisms, the secondary hairpin acts as a self-folding structure that can access complementary target double-stranded (ds) DNA, exposing priming sites for further primer hybridisation and extension at the loop regions. The topological constraints involved in the design of such motifs, including those imposed by the closure of the hairpin^6,7^, allow them to exist as long-lived functional motifs. For example, two hairpin structure-dependant methods, which require only a single enzyme to catalyse the DNA amplification reactions, have led to highly stable assays compared to other isothermal amplification methods^8^.

However, these different mechanistic methodologies have been achieved at the expense of the introduction of significant complexity in the design process, which often neglects major functional products/units as intermediates in the reactions. Consequently, many different assay methodologies have been developed, each forming a different, independent mechanism to serve the diversity of needs for such molecular detection, for DNA and RNA biomarkers, with real-time, quantitative measurement, high sensitivity or multiplexing for example.

Here we propose a more generalisable approach, in which a set of underpinning guiding principles is used to produce a method for the direct and programmable design of nucleic acid isothermal amplification assays. By combining graphical abstraction techniques with a formalism methodology, to link the primer design and amplification process with functional amplification intermediates, we provide a simplified approach to the design of enzyme-mediated isothermal amplification.

Our generic concept of the use of reaction graph abstractions for depicting hairpin and amplicon-mediated nucleic acid isothermal amplification enables us to provide strategies for creating the key functional hairpin-structure intermediate motifs that can activate the catalytic amplification steps. Although the abstraction technique, by its nature, will describe many of the existing isothermal amplification mechanisms, including LAMP, it also suggests pathways that perform assays more effectively than the commonly used systems. Importantly, the methodology also lends itself to algorithmic implementation, enabling all options to be exemplified, and prioritised.

In this paper, we demonstrate the exploration of a range of implementations of such reaction pathways, using selected case-specific systems to provide insights into mechanistic design relationships, showing, for example, how the initialisation steps are used as mediators in the subsequent amplification cycling steps, leading to the selective formation of different target products.

We also illustrate the ease and flexibility of the proposed design process by using a software tool to enable the design of primers and reactions based upon user-input target sequences. This platform demonstrates the simplicity and ubiquitous nature the reaction graph abstraction, depicting sequence information and amplification steps as different building blocks in the assay design, with the amplification process involving one reaction product as the target of a second, resulting in a cascade amplification process.

Our network model serves as a convenient method for exploring the underlying free energy of the amplification thermodynamics, enabling us to design multiplexed assay mechanisms that work across a range of challenging diagnostic tests. In one example, we show our ability to amplify and detect very short target sequences of ca. 20 nucleotides, such as miR21 and miR-7 microRNA, whose upregulation is associated with oral squamous cell carcinoma, breast cancer and glioma^9^. This demonstration meets two challenges associated with short and similar nucleic acid sequences^10^, that can be overcome simply without the need for additional extension reactions^11^, commonly required by other isothermal systems.

By working through the same prism of reaction graphs, using the ubiquitous nature of the tool, we are also able to design four sensitive, in vitro diagnostic assays, demonstrating robust and direct detection of infectious diseases from hospital samples. Each example has its own challenges, including a multiplexed test to detect HIV, with high levels of sequence variations; an assay to detect short miRNA sequences relevant in cancer diagnosis and prognosis, a highly sensitive cell-based *M. tuberculosis* assay; as well as a study for analysing patient clinical samples for hepatitis B. Although these challenges may be tackled using existing molecular designs, each requires different approaches, whilst our approach allows users to design all of the assays using the same generalisable principles.

## Results

### The concept of the reaction graph

**Fig. 1:**
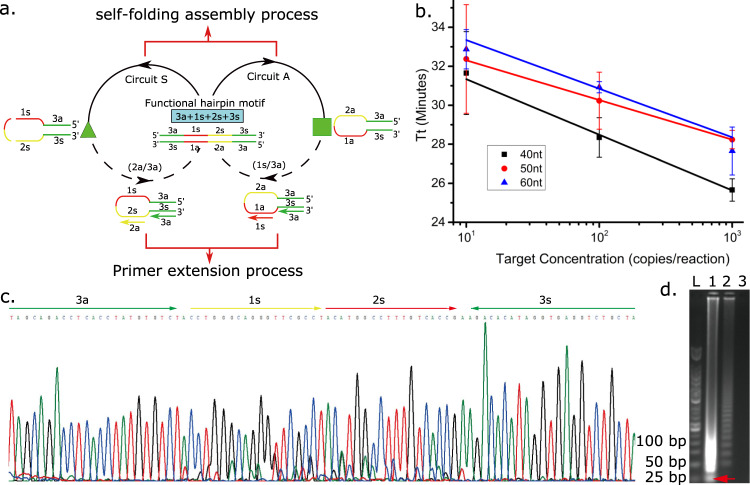
Programming a nucleic acid isothermal amplification pathway as a reaction graph. Programming a nucleic acid isothermal amplification pathway as a reaction graph in which the hairpin complex acts as the initiator and encodes two priming sites in the loop region: (**a**) A schematic for generating the secondary structure mechanism and reaction graph from the functional hairpin motif-mediated isothermal amplification. The letters a/s denote the complementarity of the nucleic acid sequence and the numbers represent specific fragments in the functional hairpin structure (e.g. 2a is complementary to 2s). During their amplification, the functional hairpin motif double strand product (blue box) disassembles (solid arrows) to form sense (green triangle) and anti-sense (green square) strand products. The formation of self-folding products exposes the priming sites which leads to the hybridisation of the primers and extension of the sequence by the strand-displacement DNA polymerase (dashed arrows) to generate a double strand amplicon. The amplicon then becomes the target (or initiator) for further amplification cycles via circuit S and A; **b** The reaction kinetics of the mechanism described in Fig. 1a with different lengths of loop fragment from 40 nt (black squares), 50 nt (red disk) and 60 nt (blue triangle). Data are presented as mean values; error bars are standard deviation, *N* = 3 independent experiments; **c** Sequencing of product (3a + 1 s + 2 s + 3 s) where the amplification products were digested (lane 1 in (**d**)), cloned and sequenced, identifying as tandem repeats; **d** Agarose gel electrophoresis of the amplification products. Lane L: 50 bp ladder; Lane 1: amplification product digested by the restriction enzyme Xba I. Lane 2: amplification product (with initiator); Lane 3: amplification product (without initiator). The gel is representative of experiments repeated independently three times with similar results. Source data are provided as a Source Data file.

Our reaction graph concept entails simple representations of functional hairpin-based nucleic acid isothermal amplification mechanisms, enabling scientists to quickly establish the main reactions and the amplicons, with the ability to design strategies addressing situations that are currently challenging when using existing methods. In order to achieve this goal and demonstrate the amplification methods for developing basic DNA nodes and circuits, the process was simplified into five components (Fig. 1a): the functional hairpin motif self-folding assembly process (solid arrows); the primer extension process (dashed arrows); the formation of functional hairpin motifs generated through amplification (blue box); numeral marked primers and functional hairpin motif self-folding products (green triangle for forward strands and green square for reverse strands).

We built the basic nodes and circuits around two core isothermal amplification reaction schemes performed using a ssDNA containing a hairpin motif (green triangle), with one priming site at the loop domain, as illustrated in Fig. 1a. The amplification process is described via the state of each port in a reaction graph^12^ and the secondary structure formation mechanism, simultaneously, also shown by Fig. 1a. As the primer that encodes the complementary sequence of the loop region (2a in circuit S and 1s in circuit A of Fig. 1a) hybridises to the loop domain, it initiates an extension reaction that opens the hairpin structure with a strand-displacing DNA polymerase (dashed arrows). The newly exposed domain (3s) at the stem serves as an initiation site that permits the generation of a double strand product, represented by the functional hairpin motif in the blue box.

Subsequently, an autonomous self-folding disassembly reaction of the functional hairpin motif occurs (solid arrows of Fig. 1a) to form two self-folding products (green triangle and green square). The formation of these intramolecular structures then separates the dsDNA product into two appropriate hairpin structures, exposing the priming sites. When primers bind to these exposed sites, the two complementary self-folding molecules serve as targets to start a new round of catalytic amplification and thus achieve signal amplification. The closure of the circuit between the blue box and triangle/square, Fig. 1, defines the continuation of the amplification process.

### Mechanistic studies

In order to understand the mechanism and thermodynamics of the self-folding of hairpin structures and the competing folding of DNA oligonucleotides with partially complementary sequences, studies were performed using melting analysis to characterise the dependence of each phenomenon with the concentration of the constituent molecules^13^. To illustrate this behaviour in our system, we explored the thermodynamics of the amplification reactions, monitoring the melting temperature, T_m_, of reactions involving oligos H1 and H2, which can both bind to each other and self-fold as hairpins individually (see Supplementary Fig. S1). Building upon our general design rules, established previously^14^, we are able to use the hairpins as a fuel for amplification reaction (noting improved stability when the neck is long, Supplementary Fig. S2).

The reaction graph, described in Fig. 1a, allows the generation of size-limited amplicons, which was confirmed by the smaller bands in Fig. 1d, showing the different structures predicted by the mechanisms as designed (and confirmed by sequencing in Fig. 1c). However, the longer structures are not predicted directly, as is often the case in isothermal amplifications (including LAMP, for example). The longer structures arise as a consequence of interactions of Bst polymerase, primers and amplification product, as described recently for LAMP using high resolution melting analysis^15^, and demonstrated here by restriction digest sequencing in Supplementary Fig. S3.

### Computational design tools

**Fig. 2:**
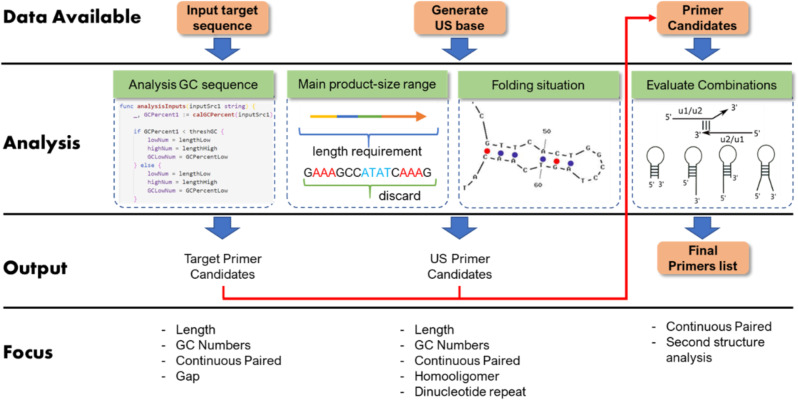
Flow chart of primers design software. Flow chart of primers design software including (1) screening target sequences, (2) randomly designing and screening US sequences, and (3) filtering and outputting the final primers according to the four different schemes (see details in Supplementary Text).

The design of functionalities into an isothermal amplification system requires significant expertise^16^ and is often an iterative approach. Our reaction graph abstraction method provides the means to enable the design of the sequences necessary for an assay, based on relationships between different sequences of building blocks, enabling us to automate the compilation of the functional motifs for hairpin-mediated isothermal amplification. We were able to demonstrate an automated primer design system which only requires as its input specific target sequence information, but which provides as an output ranked sets of primers and an isothermal amplification process with specified dynamic behaviours, Fig. 2.

We developed such a program (described in detail in the Supplementary Text) for a user to input a target sequence (e.g. from a candidate pathogen in a diagnostic question) and obtain primer sequences to enable detection through amplification. The program is extensible, designed as a flexible tool for reaction graph primer design and can meet various design requirements in a high-throughput informatics environment. It is implemented in Golang, which can be deployed on major operating systems (Windows, Linux, Mac), to achieve high throughput analysis based on multithreading, high running efficiency, native high concurrency and powerful fault-tolerance.

To simplify the primer design discussion, we refer to different primer fragments as sequences (such as 1S/2S/US), as shown in Fig. 1. As an example of the flexibility of the approach, Supplementary Fig. S4 shows how a similar reaction graph to that used in Fig. 1 can lead to a different amplification mechanism by simply removing a priming site in between the hairpin structures. The basic model of a hairpin structure-based isothermal amplification mechanism described in Fig. 1 and Supplementary Fig. S4 encompasses methods depicted for both CPA^4^ and LAMP^5^, as illustrated in Supplementary Fig. S5.

We now demonstrate the utility of these basic models by experimentally executing two different and nucleic acid isothermal amplification strategies, each of increasing complexity, and each illustrating the different mechanisms by which the interactions within the functional motifs catalyse the amplification reactions.

### Strategy 1. Generic tail strategy

**Fig. 3:**
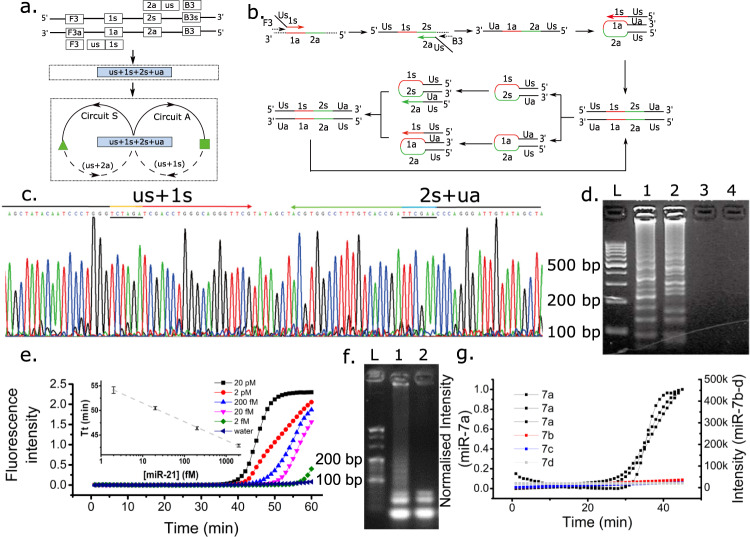
Reaction graph of isothermal amplification with primers containing a generic 5′ termini. The functional hairpin structure (us+1s+2s+ua) is formed after the target is added, starting the amplification process. **b** The secondary structure mechanism of the process described in (**a**). The primers with the generic tails are 1s+Us and 2a+Us. **c** Sequencing of functional hairpin structure (us+1s+2s+ua). (TCTAGA) and (TTCGAA) were inserted into the primers as markers. **d** Effect of template concentration on amplification. Agarose gel electrophoresis of the products as a function of target concentration. L, ladder; lane 1, 40 *M. tuberculosis* cells; lane 2, 4 *M. tuberculosis* cells; lane 3, 0.4 *M. tuberculosis* cells; lane 4, buffer (45 min reaction). The gel is representative of experiments repeated independently three times with similar results. **e**, **f** Application to the detection of mir21 microRNA (sequence 1s2s). **e** Real-time amplification curves showing concentration dependant detection for 10× serial dilutions from 20 pM (orange) to 2 fM (light blue). Negative control shown in grey. Inset is average threshold time (Tt) for different concentrations of mir-21 (*n* = 3 independent experiments, error bar is standard deviation) (**f**) Confirmation by gel electrophoresis: Lane 1 is 20 pM, 2 is blank (1 h reaction). The gel is representative of experiments repeated independently three times with similar results. **g** Specificity of the detection of miR-7 against other members of the family. (concentration: 100 fM). In black squares - three replicates of miR-7a; miR-7b (red); 5. miR-7c (grey); 6. miR-7d (blue). Left *y*-axis indicates the normalised amplification signal of miR-7a, while the right *y*-axis applies to the amplification signal of other microRNAs. Source data are provided as a Source Data file.

Our first strategy involves the design of a functional hairpin motif through sense and anti-sense primers by encoding a synthetic generic tail (termed “us”, where “u” is for “universal”) with the same sequence at their 5′ end, but with their 3′ ends complementary to the specific target sequence, Fig. 3a. The amplification pathways specified in the reaction graph of Fig. 3a was translated into the secondary structure-based molecular implementation, Fig. 3b. These primers coexist in the absence of the target. In the reaction graph, this property was programmed by the absence of a starting point (i.e. the blue box was removed from the graph). The introduction of the target led to the generation of the amplicon containing the functional hairpin motifs (us+1s+2s+ua), followed by serial self-folding and primer extension by DNA polymerase. Although not mechanistically required, we also added two outer primers (F3, B3). In a similar strategy as for their use in conventional LAMP systems, they increase the kinetics of the reactions by providing an additional route to obtain the functional amplification products, which then enable the exponential amplification phase.

Gel electrophoresis, Fig. 3d, confirmed that no amplicon was generated in the absence of the target, whilst, on addition of the target, the amplification was initiated. DNA sequencing of the amplicon revealed the expected functional motifs, Fig. 3c. This system can be used to amplify sequences from pathogens (as templates) with short, highly conserved sequences between 40 and 60 bp.

Developing assays for shorter nucleic acid sequences (down to ca. 20 nt)^17^ has been challenging using existing assay mechanisms^10^, often requiring additional steps to extend the target sequence, as is the case in Exponential Isothermal Amplification (EXPAR^18^), ligation-LAMP^11^ or RCA^19^. To this end, we demonstrate that our generic tail strategy (Fig. 3) can be directly applied to the detection of such small sequences and illustrate this capability with the detection of miR21 microRNA in a concentration dependant manner, down to fM concentrations (Fig. 3e), confirmed with a gel electrophoresis analysis (Fig. 3f). The limit of detection was estimated between 2 and 1 fM with 8 replicates, all detected at 2 fM (54.6 ± 1.2 min), whilst only half (4/8) were detected at 1 fM (56.2 ± 0.6 min) (Supplementary Table S2).

Part of the central challenge in this is that microRNAs are generally contained within families of closely related sequences with different biological functions^20^. Our mechanism was able to differentiate the miR21 from miR21-A, which differs by only 2 bases (Supplementary Fig. S6), a feat which is most usually challenging using standard isothermal assays such as LAMP of RCA^21^. We also used the wider family of miR-7 and demonstrated that the assay was able to detect miR-7-a from other members of the same family at low concentrations, often required for detection in clinical samples (Fig. 3g).

Expanding further on the capabilities of our assay design to detect small miRNA sequences, we also demonstrate that this can be carried out in a multiplexed fashion for different (but closely related) targets (Supplementary Fig. S7). The universal tail strategy can be extended with different recognition sequences, coding for different target molecules (Supplementary Fig. S7b). Existing isothermal systems have been modified to tackle this complex challenge (such as RPA^22^ or EXPAR^23^), however here we show that our strategy, involving using a universal tail, can be used to enable this design quickly and efficiently. We also demonstrate that detection can be incorporated into lateral flow strips for easy visual output (as could be required for example in resource-limited settings—Supplementary Fig. S7d).

**Fig. 4:**
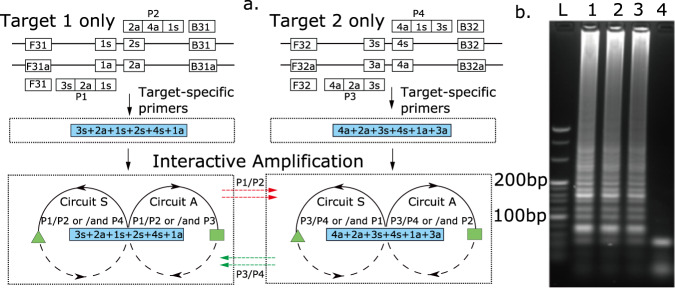
Multiplexed amplification of two HIV gene regions with cross-priming strategy. **a** Reaction graph. The primers P1-4 allow cross-amplification of 2 pathways (see Supplementary Fig. S8 for detailed functional motifs). **b** Agarose gel electrophoresis of the amplified products. Lane L—25 bp ladder; Lane 1—target 1, lane 2—target 2; lane 3—both targets 1 and 2; lane 4—no target. The gel is representative of experiments repeated independently three times with similar results. Supplementary Fig. S8 also shows the results of the sequencing of the amplification. Source data are provided as a Source Data file.

In order to further illustrate the effectiveness of multiplexing strategies, we also explored the design and implementation of multiplexed reaction graphs to detect different sequences in a single, long, nucleic acid sequence^24^ (Fig. 4), for example different regions of the same genome, in this case HIV. The design of such assays, particularly for the detection of such RNA viruses, requires to overcome their high frequency of mutations, which often results in limited conserved regions^25^. We provide a strategy whereby two sequences initiate the same analytical output, so improving the efficacy of the assay, with the amplification and diagnostic output proceeding when either one or both sequences are introduced in the assay (as might be needed for a multiplex assay when two or more variants are present).

Figure 4a shows such a design of cross primers, which contain three different constructs: (i) the 3′-end (1s in the example of P1) is complementary to the conserved region of the target (1); (ii) a sequence downstream, towards 5′ (2a of P1), complementary to a priming site on the opposite strand of the same target (2s, the priming site for P2); and (iii) an incorporated sequence (3s in P1), which is the same as the priming site of another cross primer (3s of P3) that can amplify another conserved region (2) from the target. During the amplification cycling steps, the products from the cross primers formed the functional motifs. The functional motifs from Region 1 exposed the priming sites common with Region 2, which enabled the primers for Region 2 to bind and be extended by DNA polymerase, and vice versa.

The reaction graph also enabled us to predict the sequences of the amplification product and functional motifs. In the example of HIV, we used conserved regions in the gag and pol gene. Figure 4 demonstrates the ability of the reaction to generate amplification when one, or the other, or both targets are present. The predicted sequences of the amplicons were confirmed with DNA sequencing (Supplementary Fig. S8), although similar high molecular weight structures as described in Fig. 1c are also observed.

### Strategy 2. Progressive model

**Fig. 5:**
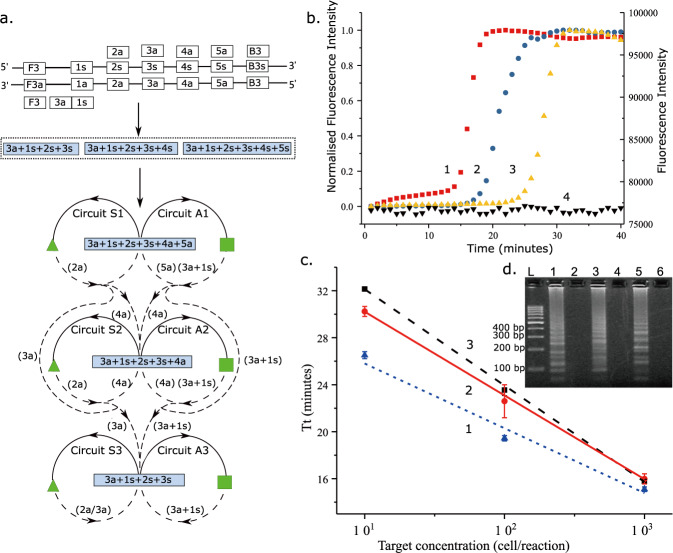
Progressive model with exponential kinetics. **a** Reaction graph. Multiple primer extension arrows entering the same circuit, mediated by (3a+1s+2s+3s, 3a+1s+2s+3s+4s, and 3a+1s+2s+3s+4s+5s). **b** Real-time amplification curve of the progressive model with 10-time serially diluted target: 1. 400 *M. tuberculosis* cells (red square); 2. 40 *M. tuberculosis* cells (blue circle); 3. 4 *M. tuberculosis* cells (yellow up triangle); 4. Negative (black down triangle); 1–3 are normalised fluorescence intensity ([0-1], left axis), whilst 4 is raw fluorescence intensity (right axis). The samples were purchased extracted genomic DNA from a reference laboratory, characterised by digital PCR (see Methods). **c** System kinetics with different number of anti-sense primers examined by real-time amplification: 1. 3a+4a+5a (blue dot); 2. 3a+4a (red solid); 3. 3a only (black dash). Threshold time (Tt) is the time corresponding to 20% of the maximum fluorescence intensity in the reaction. Data are the average of three technical replicates and error bars show the standard deviation; **d** Agarose gel electrophoresis demonstrating the effect of different numbers of anti-sense primers added to the reaction. 1/2. 3a+4a+5a; 3/4. 3a+4a; 5/6. 3a only. 1/3/5 were performed with 40 *M. tuberculosis* cells; 2/4/6 were negative. The gel is representative of experiments repeated independently three times with similar results. Supplementary Fig. S9 shows the secondary structure mechanism and the sequencing results of the product. Source data are provided as a Source Data file.

Our second strategy sought to overcome the increment in complexity in a reaction pathway, which arises when linking an additional reaction pathway onto a primary pathway, for example where one product of an amplification pathway serves as an input into a second reaction (e.g. resulting in two amplification reaction systems, as in e.g. EXPAR, the first one being linear and the second one exponential^26^). Figure 5 shows the implementation of such a step change in the complexity of design by using the intermediates as the links between different reaction pathways. In these cases, the primer extension generated many products containing functional motifs which were used as targets in the further amplification process. All pathways described here start with the addition of genomic DNA.

By programming this progressive model into the reaction graph abstraction of Fig. 5a, we obtained an isothermal amplification system with a sense primer (3a+1s) with the 3′ end complementary to the target sequence. The 5′ end was complementary to a sequence that lies downstream on the same strand of the target and anti-sense primers (2a, 3a, 4a and 5a) complementary to the individual sequences of the target, Fig. 5a. In the corresponding molecular implementation, all the primers coexisted in the absence of target molecules. The target catalyses the generation of three different functional hairpin motifs (3a+1s+2s+3s, 3a+1s+2s+3s+4s and 3a+1s+2s+3s+4s+5s) which all contained the same functional hairpin motif (3a+1s+2s+3s).

Such functional hairpin motifs tend to form intramolecular hairpin structures after self-folding and are stabilised by the paired, helical structures that form between 3a and 3s (at the 3′ end). The antisense strand product can therefore be extended after self-folding from the 3a site by adding a 4a or 4a+5a sequence at the loop region. During the primer extension, primers 2a/3a+1s bound to the loop region of sense and anti-sense strand, respectively, formed functional motifs, and led to the opening of the hairpin structure after extension, exposing the priming sites for other primers.

Critically, the formation of intermediate structures (as 3a+1s+2s+3s+4s and a hairpin motif 3a+1s+2s+3s+4s+5s) drives an exponential amplification process, powered by this progressive model. The hairpin motif (3a+1s+2s+3s+4s+5s) has two functions, namely: (i) with primers 2a/4a and 2a/3a, it catalyses the generation of 3a+1s+2s+3s+4s and 3a+1s+2s+3s in circuit S1 (and similarly in A1 with the respective primers); and (ii) with primers 2a/5a, it produces a new copy in both circuits S1 and A1.

Similarly, the motif 3a+1s+2s+3s+4s leads to the production of 3a+1s+2s+3s with the primers 2a/3a in circuit S2 (or in circuit A2 with primer 3a+1s), and a new copy of itself with the primers 2a/4a and 3a+1s/4s in circuit S2 and A2, respectively. Hence, 3a+1s+2s+3s+4s and 3a+1s+2s+3s+4s+5s form a progressive process capable of inputting another copy of the target motif to parallel amplification processes thus increasing the amplification efficiency, Fig. 5c. The amplification pathways specified in the reaction graph of Fig. 5a are thus translated into the secondary structure-based molecular implementation of Supplementary Fig. S9.

The sensitivity of this progressive model was evaluated in the design of an in vitro diagnostic assay using tenfold serially diluted target of the genomic DNA from *M. tuberculosis* cells (400 to 4 cells per reaction). Evagreen fluorescent dye was used to monitor the formation of double-stranded structures and thus characterise the kinetics of the reactions, Fig. 5b, showing faster reactions as the target concentration was increased.

The implementation of the different pathways simultaneously was gated by the presence of different anti-sense primers. Using the same real-time approach as in Fig. 5b, we defined a threshold time (Tt) and compared different reaction velocities by using a measure of the time when the fluorescence signal reaches 20% of its maximum (by analogy to the cycle threshold of real-time PCR). Figure 5c confirms that as more anti-sense primer sequences were introduced, different amplification pathways were enabled, leading to exponential reaction kinetics. Systems with 5a+4a and 4a were 13% ± 6 and 10% ± 3 respectively faster than when using 3a only, as their presence led to more opportunities to form more functional hairpin motifs. We confirmed the formation of specific functional hairpin motifs (3a+1s+2s+3s) and (3a+1s+2s+3s+4s) in the reaction graph (Fig. 5a) by agarose gel electrophoresis (Fig. 5d) and sequencing (Supplementary Fig. S9). Subsequent analysis of the amplification products by sequencing show that these large amplification products are composed of tandem repeats of the expected functional domains (the full mechanism is presented in Supplementary Fig. S9). This system can be used to amplify the DNA of pathogens with long, highly conserved sequences.

We further explored the applicability of our two de novo designs (Figs. 3a, 5a) by investigating their translation into medical diagnostics, processing 54 clinical samples from Hepatitis B virus patients, with the results shown in Table 1 (individual values are presented in Supplementary Table S3). Our methods showed the results had a high coincidence (96.3%), when compared against the real-time PCR gold standard. Only two samples were missed in these systems with copy numbers at 6.35 and 14 respectively, below the PCR clinical sensitivity threshold. As with many isothermal amplification methods^27^, our mechanisms have shown kinetics that are concentration-dependant in well-controlled systems (e.g. artificial sequences). However, clinical samples contain large amounts of inhibitors and potentially more complex sequences which limit further the applicability of the techniques as quantitative methods, for example for viral load measurements in HBC patients. In these applications qPCR should be used as the gold standard method.

**Table 1:** Comparison of the patient samples assayed for HBV by real-time PCR, as a gold standard technique used clinically, and two isothermal amplification methods. (see Supplementary Table S3 for details).

	Generic tail strategy		Progressive model	
	Pos	Neg	Pos	Neg
Real-time PCR Pos.	35	2	35	2
Neg.	0	17	0	17

## Discussion

PCR has provided the most popular amplification method for detecting low-abundance nucleic acids. However, the requirement for expensive thermal cyclers can limit its application at the point-of-care^8^. Isothermal amplification methods have overcome the need for cycling, widening the areas of application. The key technical challenge has been to open the double-stranded DNA sequences under a constant temperature to expose primer hybridisation sites, leading to the next round of amplification. Many strategies have used additional proteins and/or enzymes (on top of the polymerase activity, including ligases (in some rolling circle amplification methods, RCA), helicases (Helicase dependent amplification, HDA), nicking enzymes (Strand displacement amplification, SDA and EXPAR), RNases H (nucleic acid sequence-based amplification, NASBA) and recombinases (Recombinase polymerase amplification, RPA). Although many commercial kits are available for these reactions, their development often requires optimisation of the components (e.g. concentrations) and reaction environment (e.g. buffer composition), often achieved through iterative experimental validation^3^. Taking the example of LAMP as a comparator, our system can use fewer primers (we only optimised two primer concentrations (1–2 μM) to increase sensitivity and limit false amplification, whilst LAMP requires an additional primer optimisation process, as both FIP/BIP and LP/BP are very important for efficiency).

Our approach relies on a different strategy to open up the double-stranded molecule using functional domains, like hairpin structures (CPA) and dumb-bell structures of amplicons (LAMP). Both LAMP and CPA have previously been shown to achieve high specificity and sensitivity in a one-step reaction, by using polymerases with a strand displacement function. The central design principle of these methods lies with the formation of a functional hairpin structure contained within the amplicon, which mediates further cycling and thus amplification. In this study we demonstrated that, by using a reaction graph abstraction to specify the mechanistic steps between the self-folding disassembly and primer extension, we were able to expand upon this central principle and demonstrate the programming of complex functional hairpin structure-mediated isothermal amplification pathways, beyond LAMP and CPA. Importantly, this strategy allowed us to develop systems that can directly detect very short sequences (such as those of miRNAs) that have required complex adaptation of existing protocols. As a proof-of-concept, working in spiked buffer samples, we demonstrated the specific detection of miR21 and a multiplexed format, paving the way to compete with other strategies in clinical applications^28^.

We also directly compare our approach to LAMP, as one of the most commonly-used isothermal assay (Supplementary Fig. S10). Our mechanisms not only perform with similar kinetics (Supplementary Fig. S10a), whilst enabling wider functionalities, but also provide reaction pathways that improve both specificity (Supplementary Fig. S10b) and the reproducibility of the assay (Supplementary Fig. S10c).

In conclusion, in order to design functionalities into isothermal amplification systems, we used an underpinning generalisable methodology that is based upon a reaction graph abstraction method to enable the effective and rapid end-to-end design of the sequences necessary for a wide range of assays in a manner which, we demonstrated, can be automated. Codes and methodologies are provided on an open-source, open innovation model to enable the compilation of the functional motifs for hairpin-mediated isothermal amplification primer design using only the input specific target sequence information.

## Methods

### Ethical statement

In this study, all the procedures involving human participants were in accordance with the ethical standards of the institutional and/or national research committee. The procedures followed the 1964 Helsinki declaration and its later comparable ethical standards. All procedures were carried out in accordance with the “Measures on Ethical Review over Biomedical Research Involving Human Subjects” (Ministry of Health, China, January 11, 2007). The study was approved by the ethics review board of the First People Hospital of Linhai. All the clinical sample were collected after obtaining informed consent. The data were used in this study only. No compensation was provided. The patient characteristics are provided as Supplementary Table S4.

### Enzymes and oligonucleotides

Bst 2.0 WarmStart DNA polymerase was purchased from New England Biolabs (Shanghai, China). All oligonucleotides used (including HIV and miR targets) were synthesised and purchased from Shanghai Sangon Biological Engineering Technology and Services Co. Ltd (Shanghai, China) with the detail sequence information provided in Supplementary Table S1. For the detection of miR21 microRNA, we included LNA bases in our primers. LNA modification has been used to improve primer stability for short sequences, as well as increase the discrimination of single base differences^29^. The reverse transcription was carried out with the reverse primer without LNA modification (1 nM) with ten units (10 U) of AMV reverse transcriptase (NEB) at 37 °C for 15 mins (in 20 µl) in a buffer containing 0.8 mM of dNTP, 20 mM Tris–HCl (pH 8.8), 10 mM (NH_4_)_2_SO_4_, 10 mM KCl, 6 mM MgSO_4_, and 0.1% Triton X-100. The LNA modified primers (3 µM) and 8 U of Bst polymerase were added after this incubation step.

For all the other practical applications described here, all the functional programs start with the addition of a dsDNA target (genomic DNA). All technical replicates in this work were from different master mixes.

All the isothermal amplification systems were designed starting from a pair of outer primers (F3 and B3) based upon previous studies^5^. The outer primers support strand displacement DNA synthesis in the initial steps, thereby releasing the functional hairpin structure motif for later cycling amplification. The *T*_m_ values (calculated with our program, see Supplementary Note, or using freely available software)^30^ of the outer primers was lower than that of the other primers. They were used at 1/5–1/10th of the concentration of the other primers. When designing primers, it is important to avoid sequences that have the potential to form secondary structures. Complementarity between the primers and target are specified in the target and primer design. LNA bases were configured after primer design and melting temperatures were checked using the OligoAnalyzer software (IDT, USA)

### Multiplex systems

The amplification conditions were optimised against reaction temperature, incubation time, and concentrations of primers and Mg^2+^. The optimised reaction was carried out containing 1 µM of each of the main primers, 0.1 µM each of outer primers, 0.8 mM of dNTP, 1 M betaine (Sigma), 20 mM Tris–HCl (pH 8.8), 10 mM (NH_4_)_2_SO_4_, 10 mM KCl, 6 mM MgSO_4_, 0.1% Triton X-100, 1× Evagreen, 8 U WarmStart DNA polymerase and different amounts of template in a total volume of 20 µL. The fluorescence signal measurements of reactions were performed with a 7500 Fast Real-time PCR system (Life Technologies). The products were subjected to a 1% agarose gel electrophoresis and detected by SYBR GOLD staining. The resultant products were then isolated and purified from agarose gel using QIAquick Gel Extraction Kit (QIAGEN), and then cloned into vector pGEM-T Easy (Promega), with the insert fragment analysed by DNA sequencing. The cloned plasmids were sent to Sangon Biological Engineering Technology and Services Co. Ltd, who performed sequencing using the primers (M13).

### Multiplexing reaction conditions for HIV

The optimised reaction was carried out in a 20 µL volume containing 0.05 µM of outer primers (F31, B31, F32 and B32), 1 µM each of P1-4, 0.8 mM of dNTP, 1× isothermal reaction buffer, 1× Evagreen, 8 U *Bst* DNA polymerase 2.0 and 2 µl template DNA. The reaction mixture was incubated at 63 °C for 45 min.

### *M. tuberculosis* assay

*M. Tuberculosis* genomic DNA samples were purchased from the National Tuberculosis Reference Laboratory. The supplier extracted DNA from H37Rv bacterial culture on solid medium using the QIAamp DNA Mini Kit (QIAGEN), using ddPCR for quantification, following standard protocols. Amplification conditions were as described above.

### Translational studies with clinical samples

Fifty-four clinical samples were collected by Adicon clinical laboratories (Hangzhou, China) in March 2018. The genomic DNA was extracted from 200 µl serum using a standard magnetic beads-based extraction kit (Sansure Biotech, China). The extracted genomic DNA was measured using a quantitative PCR diagnostic kit (Sansure Biotech, Changsha, China) certified by the CFDA with a sensitivity of 20 copies per reaction, on an ABI 7500 real-time PCR system (2 min at 95 °C, followed by 45 cycles at 95 °C for 10 s and 60 °C for 30 s). The positive and negative results were defined according to the supplied instruction. For the isothermal amplification, 200 µL serum were extracted using the magnetic beads, and finally eluted in 20 µL 0.1x TE. For isothermal amplification, 5 µL of the eluted sample were taken as target. The reaction was incubated at 63 °C for 40–45 mins with the fluorescence intensity measured using the Evagreen dye. The results were analysed with real-time PCR software (LightCycler^®^ 480 Software, Version 1.5; LightCycler^®^ 96 Instrument Software. v1.2; and Applied Biosystems StepOnePlus, version 2.0, Microsoft Excel (v16.57) and plotted with OriginPro (Origin Labs, v2016). Sequencing results were visualised and analysed with Chromas (Technelysium Pty, v2.6.6).

### Reporting summary

Further information on research design is available in the Nature Research Reporting Summary linked to this article.

## Supplementary information

Supplementary Information New

Reporting Summary

## Data Availability

Source data are provided with this paper. The data generated in this study have been deposited in The University of Glasgow repository Enlighten at 10.5525/gla.researchdata.1100. Source data are provided with this paper.

## Source data

Source Data File

## Code availability

The code for the Golang Primer Program Description is available through an open GitHub repository^31^ (https://github.com/SJTUBME-QianLab/Machine_Primer_Design) and is licensed under the Open Source GNU GPLv3 Licence. The repository README.md markdown file describes the dataset and includes installation and demo instructions.
