# Supplementary material for: Programmable design of isothermal nucleic acid diagnostic assays through abstraction-based models
Source: Nat Commun. 2022 Mar 28;13:1635. doi: 10.1038/s41467-022-29101-1 (PMC8960814; doi:10.1038/s41467-022-29101-1)
Supplement: Supplementary file 1 — Supplementary Information New [file 41467_2022_29101_MOESM1_ESM.pdf]

Supplementary Information for

Programmable Design of Isothermal Nucleic Acid Diagnostic Assays through Abstraction-based Models.

Gaolian Xu, Yunfei Guo, Julien Reboud, Hao Yang, Hongchen Gu, Chunhai Fan, Xiaohua Qian, and Jonathan M. Cooper

Jonathan M. Cooper
Email: Jon.Cooper@glasgow.ac.uk; Correspondence may also be addressed to Prof. Chunhai Fan, fanchunhai@sjtu.edu.cn or Prof. Xiaohua Qian, xiaohua.qian@sjtu.edu.cn

This PDF file includes:

Supplementary Figures S1 to S12
Supplementary Tables S1 to S7
Supplementary text: Golang Primer Program

Supplementary Figures

Name	Sequence (5'-3')
H1	ACTGGACGAGCTCATTTACGATCTAGAGAGGTTGCTCAAGTGACTTTCCACGTAAATCAGCTCGTCCAGT
H2	ACTGGACGAGCTGATTTACGTGGAAAGTCACTTGAGCAACCTCTCTAGATCGTAAATCAGCTCGTCCAGT

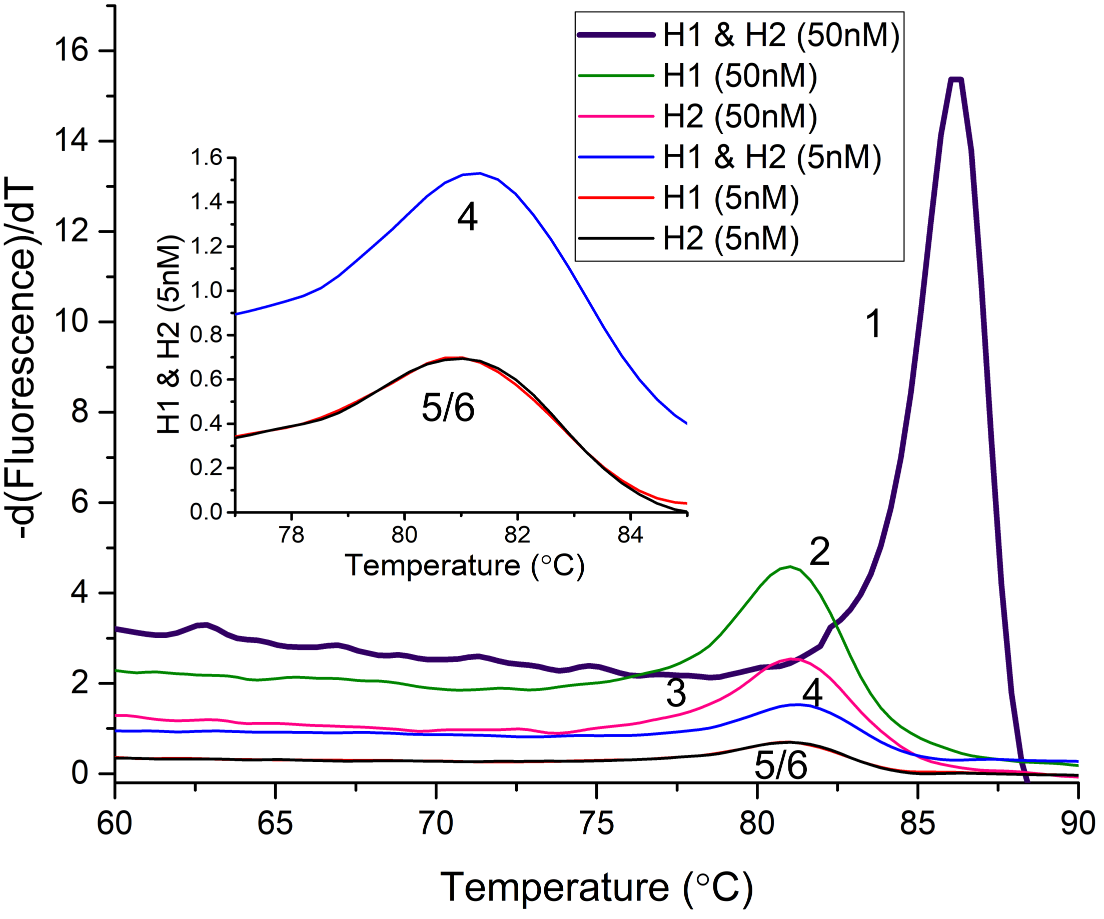

Supplementary Figure S1. DNA melting with serial dilutions of two complementary hairpin structure oligos (H1 and H2) were used for the mechanistic and thermodynamic studies of hairpin structures. The oligos concentration of 1 (dark blue), 2 (green) and 3 (pink) were 50nM, while 4 (blue), 5 (red) and 6 (black) were 5 nM, respectively. (1)/ (4). H1 & H2; (2)/ (5). H1 only; (3)/ (6). H2 only. Changes in fluorescence were acquired using standard spectroscopic methods in a temperature equilibrated cell between 95 and 55 °C at a ramping rate of 1 °C/s.

At low concentrations, 5nM, the Tm is constant when either strand is present alone or when they are both in the reaction mix, indicating a preference for self-folding. At higher concentrations, the T_m of reactions with single oligos is the same as that at low concentrations, although this shifts to a higher T_m (more stable) when the two oligos are present together, indicative of binding between them, instead of as hairpins. Source data are provided as a Source Data file.

Name	Sequence (5'-3')	ΔG (kcal.mole-1)
1	5-AGCAGACCTCACCTATGTGT ACAGCTGTACAAGACCACGTAGTCGAACTCCGCACCAGG ACACA-3	-6.13
2	5-AGCAGACCTCACCTATGTGT ACAGCTGTACAAGACCACGTAGTCGAACTCCGCACCAGG ACACATAG-3	-6.31
3	5-AGCAGACCTCACCTATGTGT ACAGCTGTACAAGACCACGTAGTCGAACTCCGCACCAGG ACACATAGGTG-3	-10.42
4	5-AGCAGACCTCACCTATGTGT ACAGCTGTACAAGACCACGTAGTCGAACTCCGCACCAGG ACACATAGGTGAGG-3	-15.61
5	5-AGCAGACCTCACCTATGTGT ACAGCTGTACAAGACCACGTAGTCGAACTCCGCACCAGG ACACATAGGTGAGGTCT-3	-19.05
6	5-AGCAGACCTCACCTATGTGT ACAGCTGTACAAGACCACGTAGTCGAACTCCGCACCAGG ACACATAGGTGAGGTCTGCT-3	-24.09

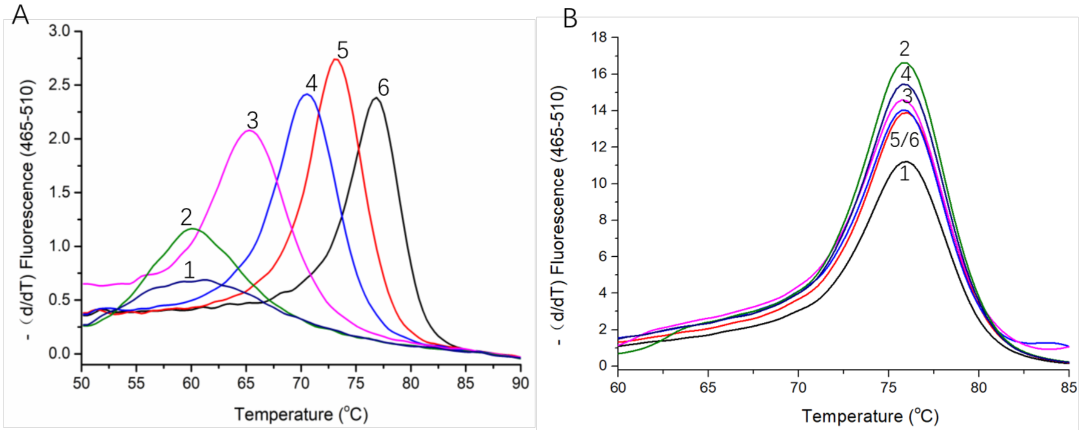

Supplementary Figure S2. DNA melting for six oligos differing in the length of stem sequence from 5 to 20 nt with the same loop size (39 nt). The hybridization kinetics gradient with six oligo designs. The oligo sequences are listed as 1 (dark blue), 2 (green), 3 (pink), 4 (blue), 5 (red) and 6 (black) with different length of complementary regions (underlined). (A) No extension; (B) self-priming after self-folding with temperature at 75 ^{o}C, folding and extension.

As the stem length decreased, the Tm also decreased reflecting the lower strength of the bonds. When the Tm values overlap in (B), the same conformations can be generated after self-priming even as the Gibbs free energy of autonomous disassembly decreased from -24.09 to -6.13 kcal/mol (using online IDT calculator). It also has been demonstrated earlier that the opening rate of hairpins is independent of loop size, whereas the closing rate is affected by the length of loop due to a lower probability of contact between the DNA ends with increasing loop size[1]. The results support a mechanism where, at the initial step, as the concentration of amplicon with hairpin structure is low, each strand of the dsDNA product exists as two intramolecular hairpin motifs, in preference over the double stranded product, due to the long complementary stem sequence (around 16 to 24 nt). This results in self-folding which then serves as template for isothermal amplification. Source data are provided as a Source Data file.

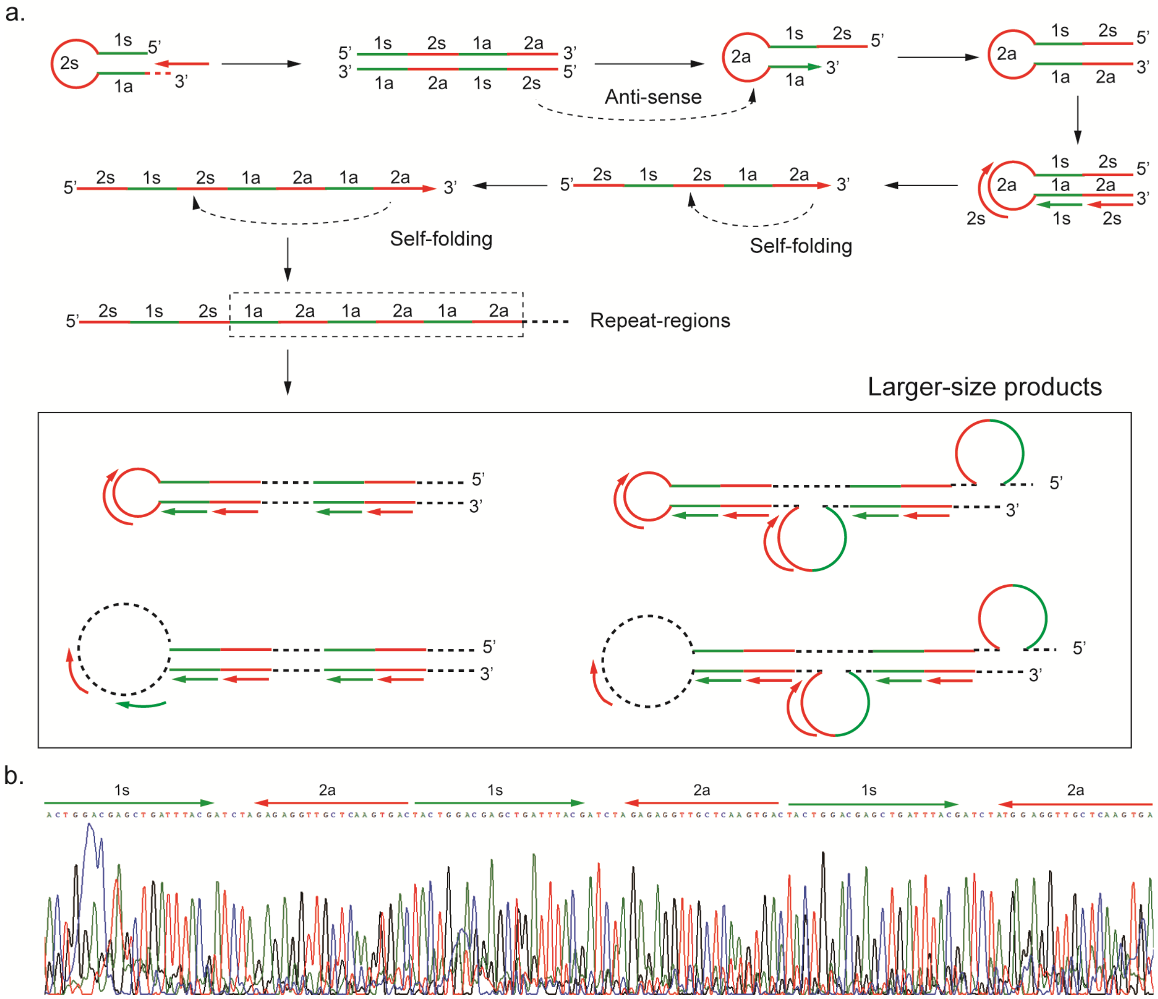

Supplementary Figure S3. Mechanisms for the formation of products with larger sizes. Forward sequences are illustrated with letter 's', and reverse with 'a'. The generation of large products arises from the extension of expected products which contains the hairpin structure, due to the non-templated synthesis ability of Bst DNA polymerase, followed by elongation to form a double strand product incorporating the primers. Based on our graph mechanism, the double strand product can, through intramolecular hydrogen bonding, form two hairpin structures. These single strands can be further extended with different cycles of self-folding and self-priming events. Repetitive cycles of self-folding and self-priming can result in the generation of products with multiple repetitive regions containing the expected sequences (dashed box) within a single molecule. These are indeed observed in our sequencing data (b) This process can give rise to very long amplicons, and a branched, mesh-like amplification network in self complementary events. The large product was initiated by the specific template sequence and contains multiple amplicon sequences, providing a high specificity and amplification efficiency, despite the fact that the end sequence of the product hosts a high level of randomness. Other potential mechanisms may include linear target isothermal multimerization and amplification, LIMA[2]. Source data are provided as a Source Data file.

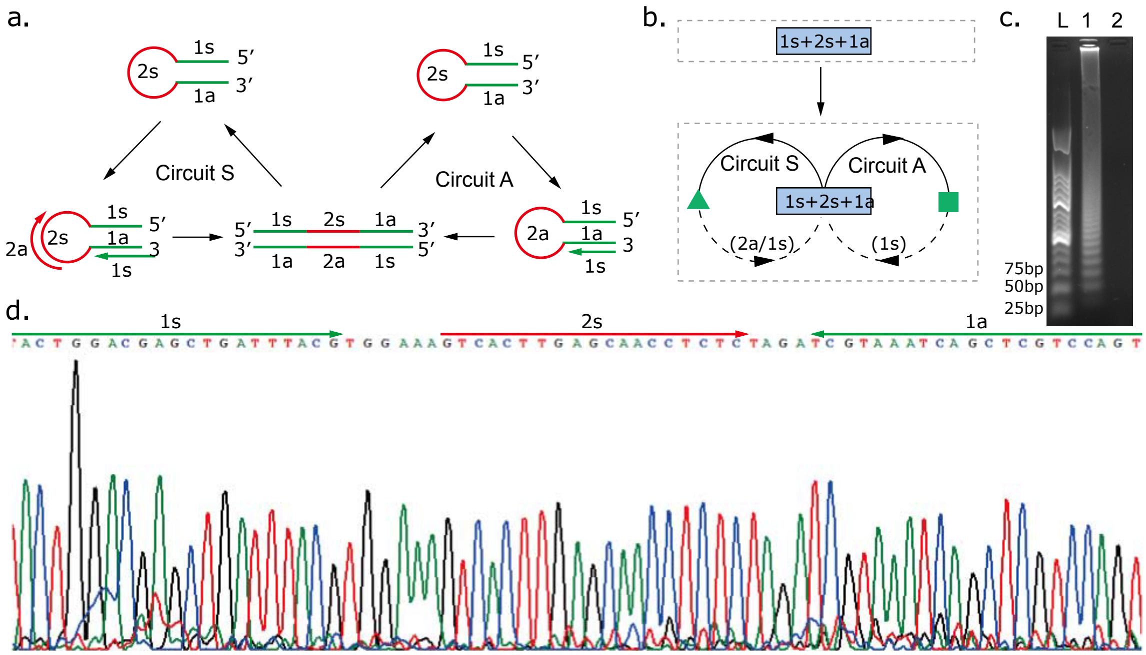

Supplementary Figure S4: A basic model of hairpin structure-based isothermal amplification in which the hairpin complex acts as the initiator and encodes one priming sites in the loop region. The ssDNA target (2s/1a/2a) can self-fold into a hairpin structure with a 20 nt stem and 30 nt loop region. Two primers (1s and 2s) are complementary to the stem and loop fragments. The products formed during the amplification were identified by DNA sequencing. (a). The mechanism illustrating the pathway during the isothermal amplification with one primer complementary to the loop region, consisting of two circuits A and S. (b). The reaction graph is based on the self-folding and primer extensions processes initiated by the presence of hairpin-shaped target (c). Agarose gel electrophoresis analysis of the amplification products. L: 25 bp ladder; Lane 1: amplification with initiator; Lane 2: amplification without initiator. (d) Sequencing of product (1s+2s+2a). The gel is representative of experiments repeated independently three times with similar results. Source data are provided as a Source Data file.

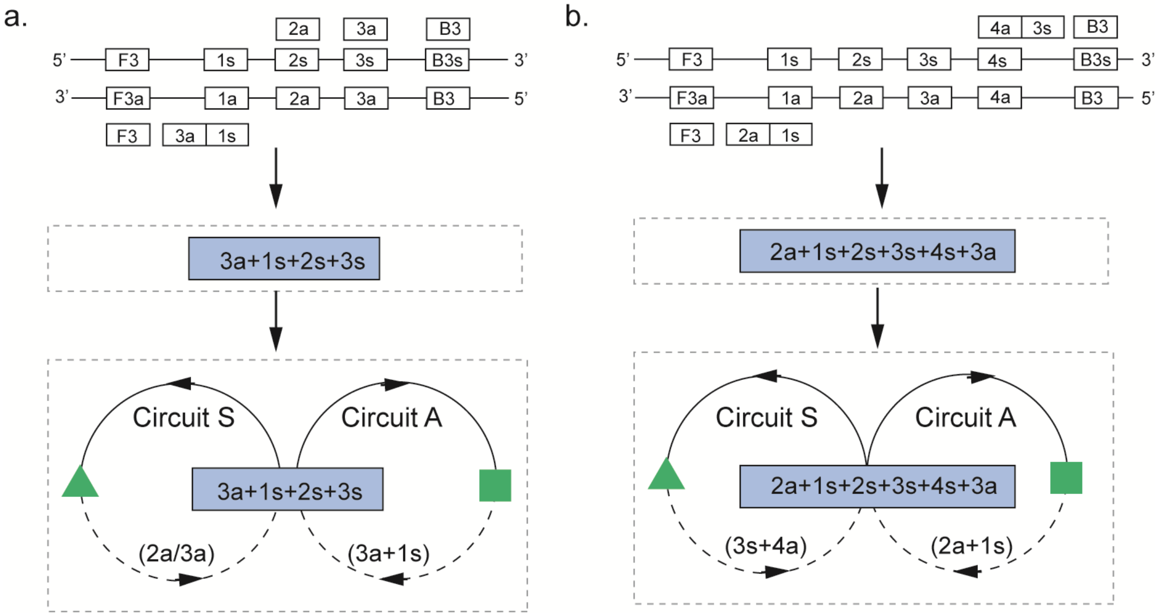

Supplementary Figure S5. Reaction graph of two previously published isothermal amplification systems: (a). CPA and (b). LAMP.

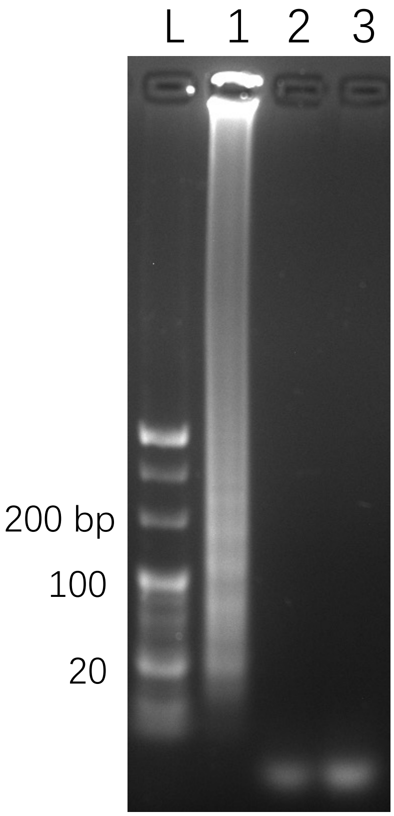

Supplementary Figure S6: miR detection specificity using gel electrophoresis of amplicons from miR21 family. Lane 1 is mir-21, 2 is mir-21-A, 3 is water as a negative control. All targets were input in the reaction at 10 fM. Lane L is 20bp ladder. The gel is representative of experiments repeated independently three times with similar results. Source data are provided in Source Data file

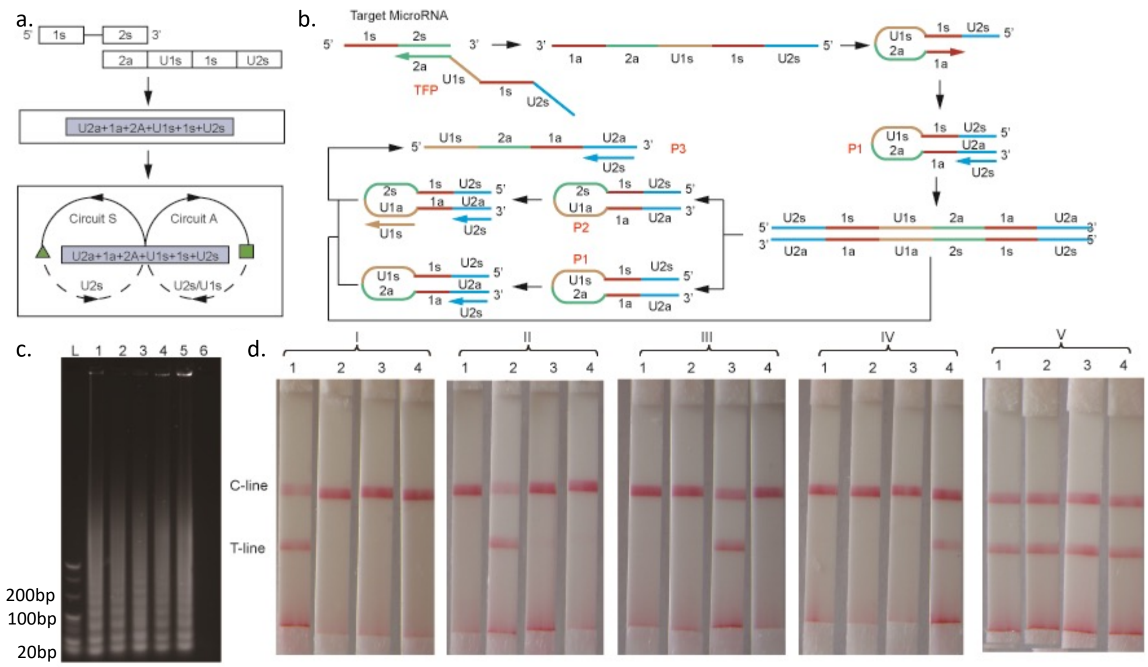

Supplementary Figure S7. miRNA multiplexing using the universal mechanism (see Figure 3 in main text). (a) Reaction graph. (b) Secondary structure mechanism. (c) Gel electrophoresis analysis with the following lanes. 1. Let 7a only; 2. microRNA 21 only; 3. microRNA 122 only; 4. microRNA 192 only. 5. Let 7a+microRNA 21+microRNA 122+microRNA 192. 6. Negative. L is a 20bp ladder. The gel is representative of experiments repeated independently three times with similar results. (d) Multiplex microRNA lateral flow detection. The template for I to V comprised all of let 7a+microRNA 21, microRNA 122, and microRNA 192. 1-4, lateral flow detection with different FITC-labeled target specific probes. 1. let 7a; 2. microRNA 21; 3. microRNA 122; 4. microRNA 192. Source data are provided as a Source Data file

Associated Methods. Reverse transcription was carried out according to the process described in the main text with four different target-specific primers (1 nM) with 100 aM of target microRNA (equivalent to ca. 300 copies). The reaction was incubated at 40 °C for 40 minutes. The products can self-fold into a complete hairpin structure through 1s and extended 1a.

Isothermal amplification was carried out according to the methods described in the main text, with 5 µL of the RT products in a total volume of 20 µL. The reaction mixture was incubated at 63 °C for 60 minutes.

The lateral flow strips (USTAR, China) have been described in details elsewhere[3,4]. In brief, hybridization products formed containing a 5' end biotin labelled primer and 3' end FITC labelled probe. The constructs bind to streptavidin and an anti-FITC antibody on the lateral flow strip. Both markers are only linked when amplification occurs, as primers are incorporated in the amplicons. Here U1s was labelled with biotin-labeled U1s, whilst to idenfify the products specifically, four FITC-labeled specific probes were added to the reaction. Supplementary Figure S7d shows specific amplification when using the multiplexed system.

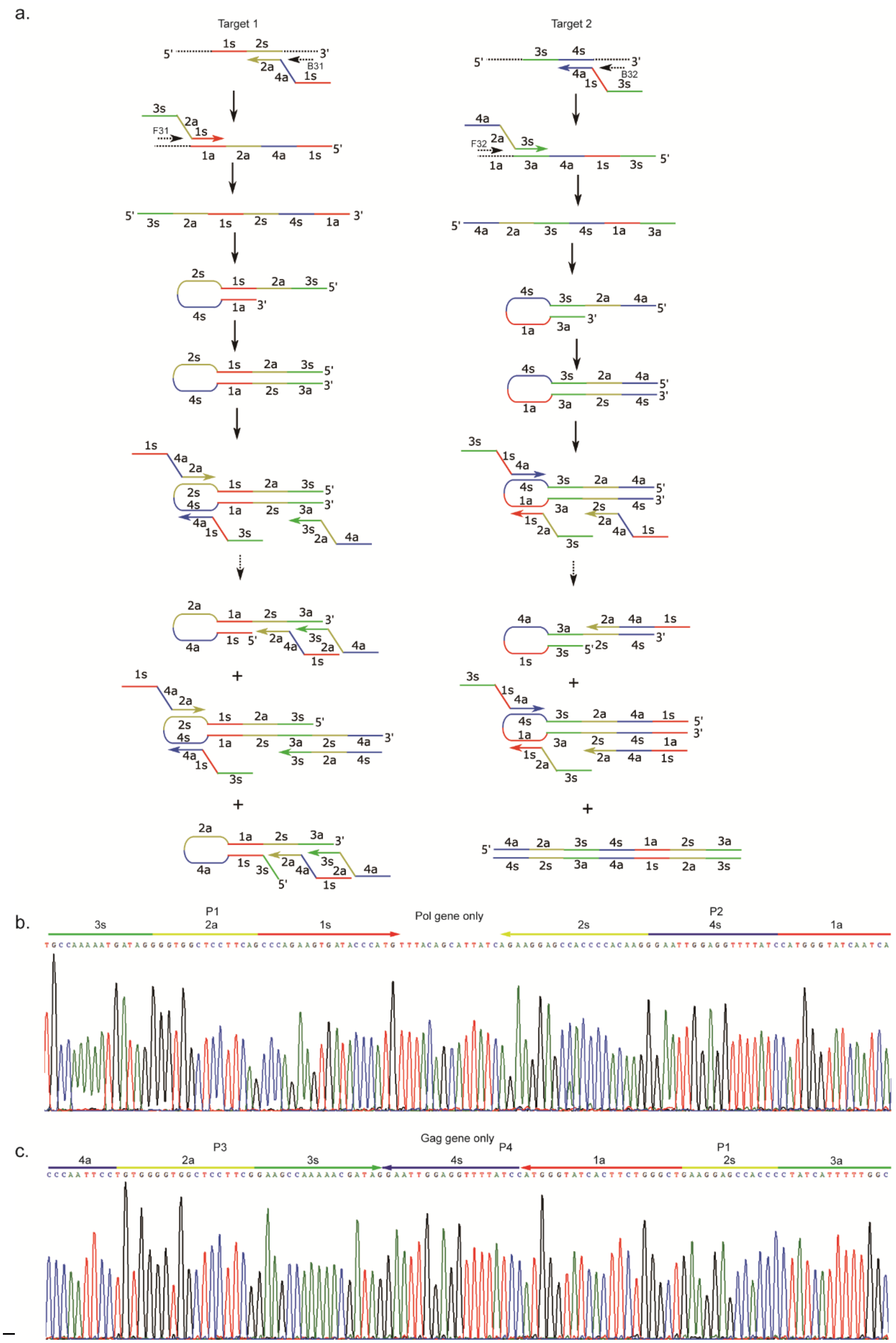

Supplementary Figure S8: (a) Detailed mechanism and DNA sequencing of the multiplexed strategy (Figure 4 main text), confirming the expected products (pol gene – (b) and gag gene bottom – (c)). Source data are provided as a Source Data file.

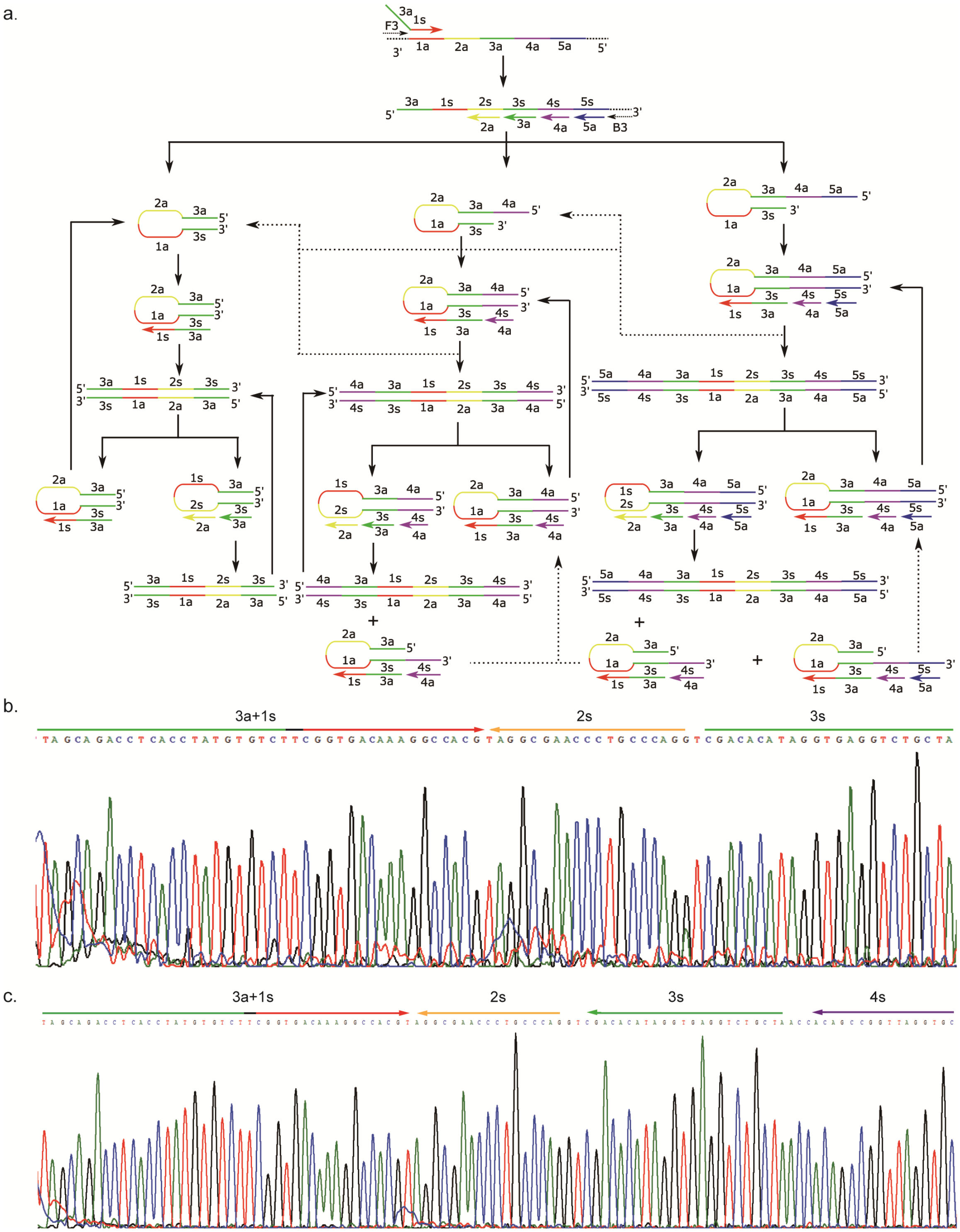

Supplementary Figure S9. (a) Secondary structure mechanism from the progressive model (Figure 5) and sequencing results showing the expected products. (3a+1s+2s+3s), (b), and (3a+1s+2s+3s+4s), (c). Source data are provided as a Source Data file.

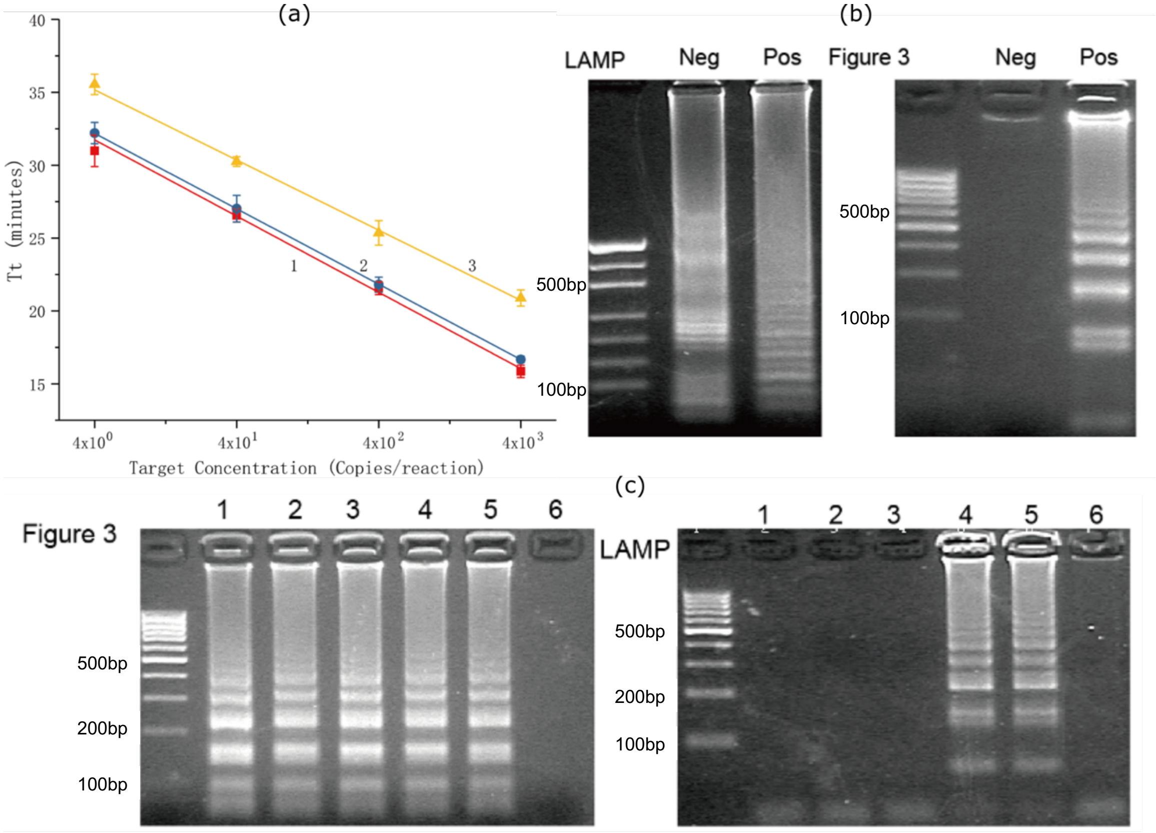

Supplementary Figure S10. (a) Kinetics of amplification for three different mechanisms, targeting the IS6110 fragment of mycobacterium tuberculosis, including: (1) Isothermal system described in Figure 5 (red square); 2. LAMP (blue circle), with primers designed using Primer Explorer v5 (see Supplementary Table S1 for sequences); 3. Isothermal system described in Figure 3 (yellow triangle). Threshold time (defined as the time corresponding to 20% of the maximum fluorescence intensity). Data is the average of 3 independent experiments and error bars represent the standard deviation. The data was fitted with linear regression (R^2>0.99). (b) Gel electrophoresis analysis of the reaction for LAMP and the mechanism of Figure 3, when the reaction is left for 90 min. In both cases, lane 0 is 100bp ladder, Neg is water and positive is the reaction containing 100 aM of target DNA. The LAMP reaction has lost specificity, whilst our mechanism still does not show unspecific amplification. We note that the negative amplification with LAMP generates a different amplicon from the expected reaction, but this would generate a positive result with many of the visualisation techniques (e.g. turbidity or Sybr Green) commonly used. We believe that this may be due to the fact that our 5' end tail in the primer reduces the opportunity of non-specific hybridization between the primers. (c) Gel electrophoresis analysis of the reaction for LAMP (right) and the mechanism of Figure 3 (left), highlighting the improved repeatability with our new mechanism for low concentrations (TB genomic DNA template at 4 copies/reaction - lanes 1-5 are positive, 6 is a negative control). (b-c) Gels are representative of experiments repeated independently three times with similar results. Source data are provided as a Source Data file.

Supplementary Table S1. Primer sequences for programming nucleic acid isothermal amplification pathways in all Figures. **Bold and underlined** bases are LNAs.

Figure No	Primer name	Sequence (5'-3')
Figure 1a	Target1	TAGCAGACCTCACCTATGTGTCTACCTGGGCAGGGTTCGCCTACGTGGCCTTTG<br>TCACCGATCTAGAAGACACATAGGTGAGGTTGCTA
	1S	CTGGGCAGGGTTCGCCT
	2A	TCGGTGACAAAGGCCACGT
	3A	TAGCAGACCTCACCTATGTGTCT
Figure 3 (a-d)	F3	ACAGCCCGTCCCGCCGAT
	B3	TGGCCATCGTGGAAGCGA
	1S	AGCTATACAATCCCTGGGTCGACCTGGGCAGGGTTCG
	2A	AGCTATACAATCCCTGGGGTCGGTGACAAAGGCCACGT
(e-f)	miR21	UAGCUUAUCAGACUGAUGUUGA
	1S	**T**A**G**C**T**T**A**T**C**A**G**A
	2A	**T**C**A**A**C**A**T**C**A**G**T**C
	Us	AGCTATACAATCCCTGGG
(g)	Let-7a	UGAGGUAGUAGGUUGUAUAGU
	Let-7b	UGAGGUAGUAGGUUGUGUGGUU
	Let-7c	UGAGGUAGUAGGUUGUAUGGUU
	Let-7d	AGAGGUAGUAGGUUGCAUAGUU
	1S	**TG**A**GG**T**A**GTAG
	2A	TTC**TATAC**AACC
	Us	AGCTATACAATCCCTGGG
Figure 5	F3	ACAGCCCGTCCCGCCGAT
	B3	TGGCCATCGTGGAAGCGA
	1S	TAGCAGACCTCACCTATGTGTCTTCGGTGACAAAGGCCACGT
	2A	ACCTGGGCAGGGTTCGCCT
	3A	TAGCAGACCTCACCTATGTGTCT
	4A	AGCACCTAACCGGCTGTGG
	5A	CAGCGCCGCTTCGGACCAC
Supplementary Figure S1	Target	ACTGGACGAGCTGATTTACGATCTAGAGAGGTTGCTCAAGTGACTTTCCACGT<br>AAATCAGCTCGTCCAGT
	1S	ACTGGACGAGCTGATTTACG
	2A	GTCACTTGAGCAACCTCTC
Figure 4	Target 1 - pol	AGTAGTAGAAGAGAAGGCTTTTAGCCCAGAAGTGATACCCATGTTTACAGCAT<br>TATCAGAAGGAGCCACCCCACAAGATTTAAACACCATGTTAAATACAGTGGGG<br>GGACATCAAGCAGCCATG
	Target 2 - gal	ATACAGGAGCAGATGATACAGTATTAGAAGAAATGAATTTGCCAGGAAGATG<br>GAAGCCAAAAATGATAGGGGGAATTGGAGGTTTTATCAAGGTGAGACAGTAT<br>GATCAAATACCCATAGAAATCTGTGGACATAAAGCCATAGGTACAGTATTAAT<br>AGGACCTACCCCTGTCAACATAAT
	F31	AGTAGTAGAAGAGAAGGC
	B31	CATGGCTGCTTGATGTCC
	P1	GCCAAAAATGATAGGGGTGGCTCCTTCAGCCCAGAAGTGATACCCATG
	P2	GATACCCATGGATAAAACCTCCAATTCCCTTGTGGGGTGGCTCCTTC
	F32	TACAGGAGCAGATGATACAG
	B32	CCTGGCTTTAATTTTACTGG
	P3	CCTCCAATTCCCTTGTGGGGTGGCTCCTTCGGAAGCCAAAAATGATAGG
	P4	AAAATGATAGG AGCCCAGAAGTGATACCCATGGATAAAACCTCCAATTCC
Supplementary	7a-TFP	CTCAATTCCGCTGCTCACG TGAGGTAGTAGG CCAGCAATCCTCCACCAAC<br>CGCAACTATACA

Figure S7

21-TFP	CTCAATTCCGCTGCTCACG TAGCTTATCAGA CCAGCAATCCTCCACCAAC CAACATCAGTT
122-TFP	CTCAATTCCGCTGCTCACG TGGAGTGTGACA CCAGCAATCCTCCACCAAC CAAACACCATT
192-TFP	CTCAATTCCGCTGCTCACG CTGACCTATGA CCAGCAATCCTCCACCAAC GGCTGTCAATT
U1s	CTCAATTCCGCTGCTCACG
U2s	CCAGCAATCCTCCACCAAC

Supplementary Table S2. Estimation of Limit of Detection for miR21. Tt (min) for 8 technical replicates at 1 and 2fM. '-' denotes no amplification.

Concentration	
2 fM	1 fM
54.76	-
56.24	55.47
53.68	-
52.92	-
56.42	-
55.16	55.85
54.33	56.84
53.31	56.95

Supplementary Table S3. Clinical study results for gold standard real-time PCR, as well as two new isothermal amplification mechanisms (Generic Tail Strategy and Progressive Model, respectively). The results where disagreement between the different techniques occur are highlighted in grey. Samples were considered negative (NEG) when no amplification was detected (no C_t for PCR, no T_t for isothermal methods). Supplementary Table S4 provides details of patients characteristics.

Sample	Copies/reaction (qPCR)	RT-PCR (Ct) (Gold standard)	Generic Tail (Tt)	Progressive (Tt)
1	NEG	NEG	NEG	NEG
2	NEG	NEG	NEG	NEG
3	1.93E+03	30.8	33.6	31.5
4	NEG	NEG	NEG	NEG
5	8.31E+02	32.0	33.4	33.3
6	6.35E+00	39.0	NEG	NEG
7	3.27E+01	36.7	37.7	37.3
8	8.32E+05	22.0	25.3	25.9
9	1.35E+03	31.3	33.9	32.2
10	2.35E+03	30.5	32.5	32.1
11	5.92E+01	35.8	37.4	34.0
12	1.78E+06	20.9	25.2	23.0
13	NEG	NEG	NEG	NEG
14	1.32E+04	28.0	30.9	30.4
15	1.38E+04	27.9	35.5	29.2

16	NEG	NEG	NEG	NEG
17	1.85E+02	34.2	35.5	29.2
18	4.34E+06	19.6	23.2	22.7
19	2.71E+03	30.3	33.6	30.4
20	8.42E+02	32.0	23.5	32.8
21	3.28E+02	33.3	34.8	34.1
22	NEG	NEG	NEG	NEG
23	5.64E+05	22.5	26.0	26.9
24	4.41E+04	26.3	29.0	27.3
25	1.40E+01	37.9	NEG	NEG
26	NEG	NEG	NEG	NEG
27	5.19E+04	26.0	30.0	29.1
28	NEG	NEG	NEG	NEG
29	2.85E+01	28.5	36.3	37.7
30	NEG	NEG	NEG	NEG
31	6.96E+01	35.6	33.6	33.0
32	NEG	NEG	NEG	NEG
33	NEG	NEG	NEG	NEG
34	1.83E+01	37.5	36.0	35.9
35	NEG	NEG	NEG	NEG
36	3.43E+03	29.9	32.2	31.8
37	4.07E+08	13.0	16.8	15.5
38	NEG	NEG	NEG	NEG
39	1.27E+02	34.7	35.6	33.1
40	2.72E+04	26.9	30.1	30.2
41	2.00E+07	17.4	20.4	18.9
42	2.70E+01	36.9	36.6	37.9
43	1.72E+05	24.3	27.0	27.5
44	NEG	NEG	NEG	NEG
45	3.10E+01	36.7	37.0	34.8
46	3.07E+01	36.8	37.9	33.6
47	5.59E+03	29.2	32.4	30.7
48	NEG	NEG	NEG	NEG
49	NEG	NEG	NEG	NEG
50	5.05E+03	29.4	31.8	31.4
51	8.32E+03	28.6	31.4	30.8
52	1.18E+03	31.5	34.0	32.9
53	1.35E+02	34.5	36.1	30.3
54	NEG	NEG	NEG	NEG

Supplementary Table S4. Aggregated patients characteristics for samples used in the study with clinical samples. Gender (male; female) is based on biological sex characteristics and was self-reported.

Gender	Male	27
	Female	27
Age (years old)	⩽50	7
	51-60	25
	61-65	11
	≥66	11
Height (cm)	⩽155	16
	156-160	12
	161-170	9
	≥171	17
Weight (kg)	⩽55	16
	56-60	8
	61-70	8
	71-75	16
	≥71	6
Medication history	Y	16
	N	38
Past surgical history	Y	3
	N	51

Supplementary Information Text

Golang Primer Program Description.
In this work, we developed an extensible program designed as a flexible tool for reaction graphic primer design.
To simplify the primer design discussion, we refer to different primer fragments as sequences mentioned in the different mechanisms in the manuscript (such as 1S/2S/US of Figure 3 main text for example), as shown in Supplementary Figure S11 below. To be consistent with earlier studies, all sequences in this paper are read in the 5' to 3' direction.

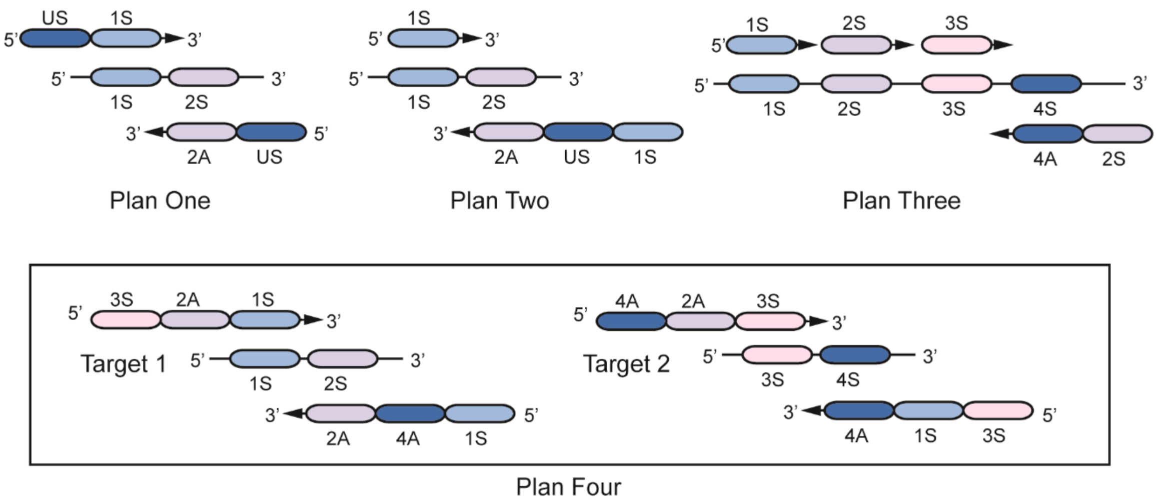

Supplementary Figure S11. Descriptions of different sub-primer fragments under different output schemes.

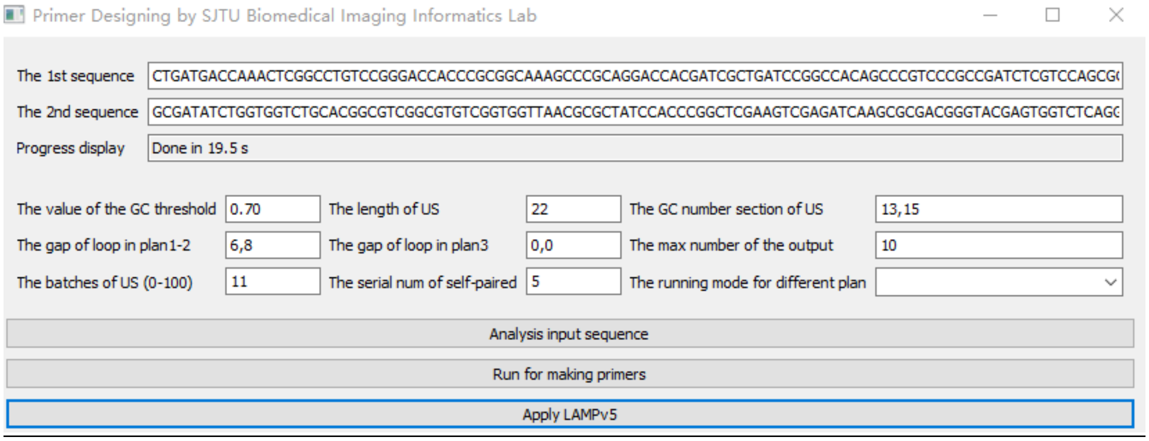

Supplementary Figure S12. GUI interface of reaction graph primer designing software. The program first screens the target sequences, designs and screens US sequences, through automatic continuous fragmentation analysis, constrained by the requirements of the mechanism of the reaction graph. To be consistent with earlier studies, all sequences in this paper are read in the 5' to 3' direction.

The parameters represented by the input box in the program indicate:

"The 1st sequence" :	Used to input the first target sequence ;
"The 2nd sequence" :	Used to input the second target sequence ;
"Progress display" :	Used to show program run-time progress ;
"The value of the GC threshold" :	Used to define the threshold for judging whether the target sequence is rich in GC bases ;
"The input length of US" :	Used to define the length of the randomly generated US sequence ;
"The GC number section of US" :	Used to define the content of GC bases in US ;
"The gap of loop in plan1-2" :	Used to define the minimum distance between paired bases in complementary paired sequence

“The max number of the output” :	Used to define the number of final primers to be exported. Once the exported primers reach this threshold, the program automatically exit ;
“The batches of US” :	Used to define the thread for screening the US sequence;
“The serial number of self-paired” :	Used to define the maximum length of consecutive paired bases in the final output primer
“The running mode for different plan”:	Used to select running mode, mode-one corresponding to scheme 1, 2, and 3, and mode-two corresponding to scheme 4

In addition, "Analysis input sequence" button was used to start a continuous segmentation analysis of the target sequence to find potential primer fragments. "Run for making primers" button was used to start screening the combinations of different sequences. The results were displayed in the log box and saved locally as a TXT file.

To enable direct comparison of the functionality of the software with other available platforms, we also included a tab called “LAMPv5”, in which our software outputs primer sequences for this specific mechanism (described by our processes in Figure 5b in the main text). We have compared the outputs to PrimerExplorer and have not seen differences for the targets we have used in this study.

1. Target Sequences Screening

First, the inputted target sequence was traversed to generate all possible combinations of primer sequence fragments. In this traversal process, based on the primer amplification technology's design rules, the program parameters were individually customized to filter out invalid primer combinations in the target sequence. In addition to Supplementary Table S5 shows the default primer design parameters of our program. Specifically, the GC content in the target sequence was automatically analyzed, and then the length and position of the sequence fragments were restricted according to whether the target sequence is rich in GC bases (GC content > 70%). A maximum poly-base restriction was enforced to limit the number of consecutive complementary paired bases in the potential primer region (complementary sequences are defined as symmetric sequences, such as 5'-CCCGGG-3' and 5'-GAATTC-3') during the filtering period based on continuous segmentation analysis. Moreover, complementary pairing can occur both within the sequence fragment itself and between two different sequence fragments. Subsequently, the above restriction processes were carried out separately for the complementary pairing within the sequence fragment and the complementary pairing between two different sequence fragments.

Supplementary Table S5 The default primer design parameters for screening target sequences

Parameter	Default Target	
	GC>=70%	GC <70%
1S/2S length	18-22bp	20-25bp
G/C number	>11bp	>8bp
Continuous self-paired length in 1S/2S	<4bp	<4bp
Continuous paired length between 1S & 2A	<4bp	<4bp
1S+2S length	<=50bp	<=50bp
Continuous self-paired length in 3S/4S	<4bp	<4bp
Continuous paired length between 3S & 2A	<4bp	<4bp
Continuous paired length between 4S & 2A	<4bp	<4bp
Gap between 1S & 2S	<=10bp	<=10bp
Gap between 3S & 4S	<=10bp	<=10bp

2. US Sequences Designing and Screening.

During the enumeration of possible US sequences, the system generates randomly designed primers to provide full diversity, which may contain partial complementary sequences or 'special' sequences (structures such as homo-oligomers and dinucleotide repeats). More importantly, these special sequences can form primer-dimers, so they were strictly excluded. The length and base content of sequence fragments was limited to control the number of primer combinations and eliminate special sequences with potential risks. Specifically, the program will check the risk of each sequence fragment, forming a complementary pair. For special sequences that may form primer-dimers, the corresponding rules and restrictions were also be deployed to exclude risky sequences strictly. Finally, the randomly designed primers may form base aggregation (such as long continuous A/T or G/C base fragments), and corresponding parameters are also established in the program to limit the length of aggregated base fragments. Supplementary Table S6 lists the default primer design parameters related to this section.

Supplementary Table S6 The default primer design parameters for screening US sequences

Parameter	Default Target
US length	22-24bp
G/C number	13-15bp
Homooligomer length (such as AAA, or CCC)	<3bp
Dinucleotide repeat length (such as ATAT)	<5bp
Continuous self-paired length	<4bp
Continuous AT/GC length	<7bp

3. Final Primers Filtering and Outputting

The potential primers from the target sequence and US primer candidates are combined to primers candidates with the different output schemes, and then evaluated to produce final primers (US+1S US+2A, 1S, 1S+US+2A, 4S, 3S, 1S+2A, 3S+2A+1S, 1S+4A+2A, 4A+2A+3S, 3S+1S+4A). In these schemes, the combination of different sequence fragments brings the risk of introducing special sequences; thus, each sequence fragment's complementarity should be rechecked. The complementarity check is still performed through the two perspectives of self-complementary pairing and complementary pairing between different sequences.

The primer sets outputted by the program are ranked in order of best prediction the delta G value, following by the formation of self-hairpin structure. We compare the delta G value of primer self-hairpin structures: the bigger the differences, the better the primers are. We provide the top five groups.

Supplementary Table S7 shows the detailed parameter settings. Although these numerous conditional screening processes extend a single program's processing time, the operating efficiency of this program is still guaranteed through multithreaded parallel operations. Where required (e.g. for short target sequences), the user can modify the sequences with the addition of special bases (such as LNA in our Figure 3 – main text). The melting temperature of the modified primers can be checked using existing programs (such as OligoAnalyzer, from IDT)

Supplementary Table S7 The detailed parameter settings for primers combination

Parameter	Default Target
Continuous self-paired length in US+1S/US+2A	<4bp
Continuous paired length between 1S & 1S+US+2A	<5bp
Continuous self-paired length in 1S+US+2A	<4bp
Continuous self-paired length in 1S+2A	<4bp
Continuous paired length between 1S & 1S+2A	<5bp
Continuous paired length between 3S & 1S+2A	<5bp
Continuous paired length between 4S & 1S+2A	<5bp
Continuous self-paired length in 3S+2A+1S	<4bp
Continuous self-paired length in 1S+4A+2A	<4bp
Continuous self-paired length in 4A+2A+3S	<4bp
Continuous self-paired length in 3S+1S+4A	<4bp
Continuous paired length between 1S/2A/3A/4A	<5bp

Supplementary references

1. Bonnet, G., Krichevsky, O. & Libchaber, A. Kinetics of conformational fluctuations in DNA hairpin-loops. *PNAS* **95**, 8602–8606 (1998).
2. Hafner, G. j., Yang, I. c., Wolter, L. c., Stafford, M. r. & Giffard, P. m. Isothermal Amplification and Multimerization of DNA by Bst DNA Polymerase. *BioTechniques* **30**, 852–867 (2001).
3. Reboud, J. *et al.* Paper-based microfluidics for DNA diagnostics of malaria in low resource underserved rural communities. *PNAS* **116**, 4834–4842 (2019).
4. Guo, X. *et al.* Smartphone-based DNA diagnostics for malaria detection using deep learning for local decision support and blockchain technology for security. *Nat Electron* **4**, 615–624 (2021).
