# Supplementary material for: Programmable design of isothermal nucleic acid diagnostic assays through abstraction-based models
Source: Nat Commun. 2022 Mar 28;13:1635. doi: 10.1038/s41467-022-29101-1 (PMC8960814; doi:10.1038/s41467-022-29101-1)
Supplement: Supplementary file 2 — Reporting Summary [file 41467_2022_29101_MOESM2_ESM.pdf]

nature research

Corresponding author(s): Jonathan M. Cooper; Chunhai Fan; Xiaohua Qian

Last updated by author(s): Feb 7, 2022

Reporting Summary

Nature Research wishes to improve the reproducibility of the work that we publish. This form provides structure for consistency and transparency in reporting. For further information on Nature Research policies, see our Editorial Policies and the Editorial Policy Checklist.

Statistics

For all statistical analyses, confirm that the following items are present in the figure legend, table legend, main text, or Methods section.

n/a	Confirmed	
☐	☒	The exact sample size (*n*) for each experimental group/condition, given as a discrete number and unit of measurement
☐	☒	A statement on whether measurements were taken from distinct samples or whether the same sample was measured repeatedly
☒	☐	The statistical test(s) used AND whether they are one- or two-sided<br>*Only common tests should be described solely by name; describe more complex techniques in the Methods section.*
☒	☐	A description of all covariates tested
☒	☐	A description of any assumptions or corrections, such as tests of normality and adjustment for multiple comparisons
☐	☒	A full description of the statistical parameters including central tendency (e.g. means) or other basic estimates (e.g. regression coefficient) AND variation (e.g. standard deviation) or associated estimates of uncertainty (e.g. confidence intervals)
☒	☐	For null hypothesis testing, the test statistic (e.g. *F*, *t*, *r*) with confidence intervals, effect sizes, degrees of freedom and *P* value noted<br>*Give P values as exact values whenever suitable.*
☒	☐	For Bayesian analysis, information on the choice of priors and Markov chain Monte Carlo settings
☒	☐	For hierarchical and complex designs, identification of the appropriate level for tests and full reporting of outcomes
☒	☐	Estimates of effect sizes (e.g. Cohen's *d*, Pearson's *r*), indicating how they were calculated

Our web collection on statistics for biologists contains articles on many of the points above.

Software and code

Policy information about availability of computer code

Data collection	The real-time amplification curves were obtained using commercial real-time PCR machine software (LightCycler® 480 Software, Version 1.5; LightCycler® 96 Instrument Software. 1. 2; Applied Biosystems StepOnePlus, version 2.0)
Data analysis	Microsoft Excel 365 was used for analysis of amplification curves, with results plotted using Origin (Origin Labs, v2016). Sequencing data was analysed using with Chromas (Technelysium Pty, v2.6.6).<br>The code for the Golang Primer Program Description is available through an open GitHub repository31 (https://github.com/SJTUBME-QianLab/Machine_Primer_Design)

For manuscripts utilizing custom algorithms or software that are central to the research but not yet described in published literature, software must be made available to editors and reviewers. We strongly encourage code deposition in a community repository (e.g. GitHub). See the Nature Research guidelines for submitting code & software for further information.

Data

Policy information about availability of data

All manuscripts must include a data availability statement. This statement should provide the following information, where applicable:

- Accession codes, unique identifiers, or web links for publicly available datasets
- A list of figures that have associated raw data
- A description of any restrictions on data availability

The data generated and analysed during the current study are available on The University of Glasgow repository (http://dx.doi.org/10.5525/gla.researchdata.1100).

Field-specific reporting

Please select the one below that is the best fit for your research. If you are not sure, read the appropriate sections before making your selection.

☒ Life sciences ☐ Behavioural & social sciences ☐ Ecological, evolutionary & environmental sciences

For a reference copy of the document with all sections, see nature.com/documents/nr-reporting-summary-flat.pdf

Life sciences study design

All studies must disclose on these points even when the disclosure is negative.

Sample size	No sample-size calculation was performed. However We aimed to recruit 50 patients, including at least 15 negative patients to establish a robust analytical comparison with PCR gold standard. During recruitment, we obtained 4 more samples that we decided to use as it would only increase confidence in the results.
Data exclusions	No data were excluded from the analyses.
Replication	Data was replicated, and comparisons between replicates are shown in Figures 1 to Figure 5, and supplementary Figures S1-S10.
Randomization	The samples were not randomized. They were processed in the order they were obtained, which is random with respect to covariates such as viral loads
Blinding	The investigators were blinded to group allocation during sample collection and analysis for the studies presented in Table 1. All other studies of an analytical nature were not carried out in a blind manner, but use biological replicates and regression analysis served to ensure minimal experimental bias.

Reporting for specific materials, systems and methods

We require information from authors about some types of materials, experimental systems and methods used in many studies. Here, indicate whether each material, system or method listed is relevant to your study. If you are not sure if a list item applies to your research, read the appropriate section before selecting a response.

Materials & experimental systems

n/a	Involved in the study	
☒	☐	Antibodies
☒	☐	Eukaryotic cell lines
☒	☐	Palaeontology and archaeology
☒	☐	Animals and other organisms
☐	☒	Human research participants
☒	☐	Clinical data
☒	☐	Dual use research of concern

Methods

n/a	Involved in the study	
☒	☐	ChIP-seq
☒	☐	Flow cytometry
☒	☐	MRI-based neuroimaging

Human research participants

Policy information about studies involving human research participants

Population characteristics	The participants were 45-75 years old, half male and half female. The study focused on analytical characterization, so the numbers of participants were not specified to disaggregate these characteristics.
Recruitment	All the samples were collected when the participants came to the hospital and were prescribed a related clinical test. This potentially biais the sample numbers to more positive samples (as patients are symptomatic, but we ensured enough negative samples were obtained by staging recruitment. It could also bias viral load towards higher values, which could inflate performance. However samples were spread widely, including for example 22 samples with Ct (PCR) above 30, indicating low viral loads. We also observed two samples that were not detected (Ct>37) which provides confidence in the robustness of the analysis.
Ethics oversight	The study was approved by the ethics review board of the First People Hospital of Linhai

Note that full information on the approval of the study protocol must also be provided in the manuscript.
